# Bacterial genome engineering and synthetic biology: combating pathogens

**DOI:** 10.1186/s12866-016-0876-3

**Published:** 2016-11-04

**Authors:** Malathy Krishnamurthy, Richard T. Moore, Sathish Rajamani, Rekha G. Panchal

**Affiliations:** Department of Target Discovery and Experimental Microbiology, Division of Molecular and Translational Sciences, U. S. Army Medical Research Institute of Infectious Diseases (USAMRIID), Fort Detrick, Frederick, MD 21702 USA

**Keywords:** Synthetic Biology (SB), Multidrug resistant (MDR) pathogens, Antibiotic resistance, Genome engineering, Antibacterial, Quorum sensing, Gene circuits, Pathogenesis, Recombineering, Targetron

## Abstract

**Background:**

The emergence and prevalence of multidrug resistant (MDR) pathogenic bacteria poses a serious threat to human and animal health globally. Nosocomial infections and common ailments such as pneumonia, wound, urinary tract, and bloodstream infections are becoming more challenging to treat due to the rapid spread of MDR pathogenic bacteria. According to recent reports by the World Health Organization (WHO) and Centers for Disease Control and Prevention (CDC), there is an unprecedented increase in the occurrence of MDR infections worldwide. The rise in these infections has generated an economic strain worldwide, prompting the WHO to endorse a global action plan to improve awareness and understanding of antimicrobial resistance. This health crisis necessitates an immediate action to target the underlying mechanisms of drug resistance in bacteria.

**Research:**

The advent of new bacterial genome engineering and synthetic biology (SB) tools is providing promising diagnostic and treatment plans to monitor and treat widespread recalcitrant bacterial infections. Key advances in genetic engineering approaches can successfully aid in targeting and editing pathogenic bacterial genomes for understanding and mitigating drug resistance mechanisms. In this review, we discuss the application of specific genome engineering and SB methods such as recombineering, clustered regularly interspaced short palindromic repeats (CRISPR), and bacterial cell-cell signaling mechanisms for pathogen targeting. The utility of these tools in developing antibacterial strategies such as novel antibiotic production, phage therapy, diagnostics and vaccine production to name a few, are also highlighted.

**Conclusions:**

The prevalent use of antibiotics and the spread of MDR bacteria raise the prospect of a post-antibiotic era, which underscores the need for developing novel therapeutics to target MDR pathogens. The development of enabling SB technologies offers promising solutions to deliver safe and effective antibacterial therapies.

## Background

The emergence of multidrug resistant (MDR) pathogenic organisms has become an important national and global health challenge. Importantly, the evolution of bacterial MDR pathogens is rampant and needs immediate countermeasures to limit lasting damage. According to the World Health Organization’s (WHO) report in 2014 on global surveillance of antimicrobial resistance, the increased rise in MDR pathogenic bacteria is putting at risk the ability to treat common ailments such as urinary tract infections, pneumonia and bloodstream infections globally, that were readily treatable for decades. In 2015, the 68th World Health Assembly has endorsed a global action plan to improve awareness and understanding of antimicrobial resistance [1]. This plan calls for the development of new medicines, diagnostic tools, vaccines and other interventions to ensure continued treatment and prevention of infectious diseases caused by bacteria. Therefore, there is an urgent need for understanding drug resistance mechanisms in MDR pathogens and targeting these mechanisms (e.g., antibiotic target site mutation, efflux pump for antibiotic expulsion, etc.) to tackle these pathogens. In addition, identification of novel and improved therapeutic small molecules and metabolic engineering for the production of these small molecules is another approach to assuage drug-resistance in MDR pathogens.

Recent advancements in synthetic biology (SB) have enabled the development of novel genome engineering tools for the manipulation of microbial genomes for various biotechnological and biomedical applications [2–6]. SB offers a novel platform to bridge the gap between basic and translational research and has the potential for providing innovative solutions to combat infectious agents. SB is an emerging field which combines engineering principles and biological parts to design novel, modular and tunable gene products or genetic circuits for modification of existing biological systems. In addition to building gene circuits for desired cellular function or metabolic engineering, there is a growing interest among synthetic biologists to develop microbial genome engineering tools. Precise changes in the bacterial genome have resulted in the creation of useful biological traits in engineered strains. The marriage of genome engineering tools and SB has further enabled the use of engineered bacteria to address some of the global challenges spanning renewable energy to global health. In particular, the recent advances in bacterial genome engineering methods that can target broad range of bacterial hosts has opened new avenues for fighting bacterial infections [7, 8]. The development of new SB tools should pave the way for developing novel approaches to address the imminent threat with antibiotic resistance in bacteria.

SB applications in bacteria have broadly ranged from building small gene circuits for a desired gene/pathway function to engineering the whole genome [9–12]. A number of investigations employing SB have also offered insights into antibiotic resistance mechanisms. For instance, to delineate the mechanism of resistance to a particular antibiotic, lethal concentrations of antibiotic triclosan was used in *Escherichia coli* to identify the gene candidates that were involved in triclosan resistance [13]. An overexpressed genomic library was generated in triclosan enriched media and using a DNA microarray, the genes that enabled the growth of *E. coli* in the presence of triclosan were identified and validated by overexpressing the candidate gene in bacteria [14]. Unlike traditional methods, which involve genome sequencing to identify potential genes that confer antibiotic resistance, this approach utilized genome libraries cloned into plasmids for expression in bacteria and enrichment in the presence of antibiotic. Such SB approach allows for genome wide screening and identification of genes as well as the effect of overexpression of these genes on cellular fitness. This is particularly useful for understanding the complex mechanisms of antibiotic resistance and for identifying one or multiple gene targets that lead to resistance. Similarly, SOS response systems in *E. coli* subjected to other antibiotics have been examined by building gene circuits in *E. coli* to study DNA damage and to understand the role of these systems in antibiotic resistance [15].

Minimal bacterial genomes have been synthesized using top-down and bottom-up approaches for identifying the essential genes in bacteria (*E. coli*, *Pseudomonas putida, Mycoplasma*) for potential therapeutic targeting [16–18]. The growth potential and the impact of SB in countering antibiotic resistance are clearly evident from these examples. The focus of this review is to highlight the applications of bacterial genome engineering and synthetic biology tools in targeting emergent bacterial pathogens and further discuss the utility of SB in advancing novel antibacterial therapeutics.

## Main text

### Genome engineering tools and their applications for countering bacterial infections

**Fig. 1:**
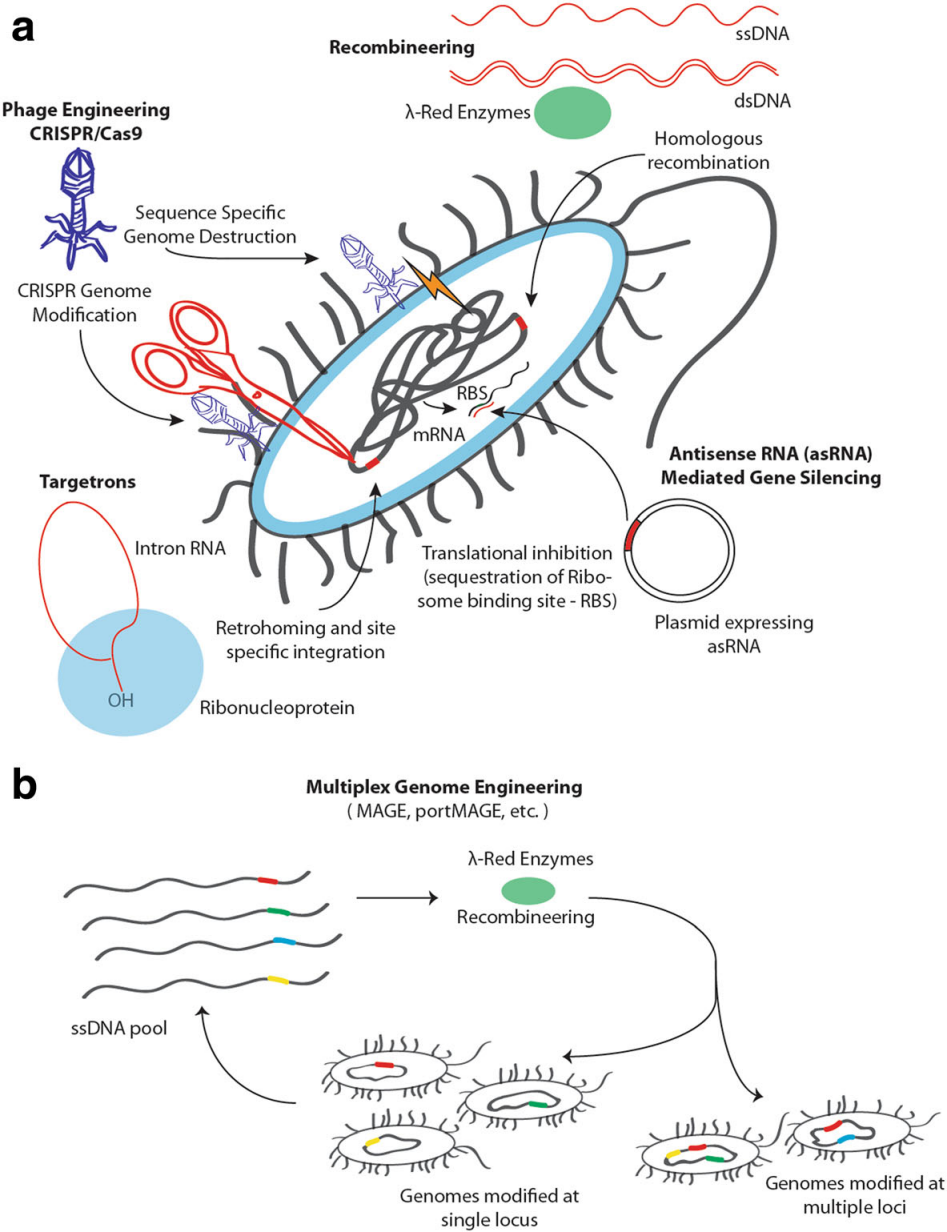
**a**) Schematic of genome engineering tools developed in *E. coli* that have been expanded to broad bacterial hosts **b**) Multiplexed Automated Genomic Engineering (MAGE) for modifying bacteria at multiple genomic loci

A number of methods have been developed for engineering bacterial genomes with varying degrees of efficiency, specificity and broad host applicability [19]. Most often, the bacterial genome editing is carried out to knock-out genes, knock-in genes or introduce mutations in the bacterial genome. Though most of these methods were developed in *E. coli*, in the last decade there has been a rapid development and expansion of these tools to a broad range of bacterial hosts (Fig. 1a). Noteworthy is the tractability of these engineering tools in other pathogenic bacteria, paving the way for exploration and understanding of these pathogens for combating bacterial infections. A number of useful reviews have also detailed the principles and techniques of bacterial genome engineering tools [7, 8]. The most common tools that are currently being utilized for genome engineering of pathogenic bacteria are summarized in Table 1 and their potential applications in countering bacterial infections are discussed below.

**Table 1:** Tools available in pathogenic Gram-negative and Gram-positive bacteria for genome modification

Engineering tool	Methodology	Engineered pathogenic strains
Recombineering	Homologous recombination of linear DNA utilizing λ-Red enzymes Gam, Exo and Bet	*Salmonella enterica* [26] ^a^ *Escherichia coli* [23] ^a^ *Pseudomonas aeruginosa* [29] ^a^ *Streptomyces coelicolor* [28] ^b^ *Shigella dysenteriae* [50] ^a^
pORTMAGE	Portable Multiplex Automated Genome Engineering (MAGE) Utilizing recombineering	*Salmonella enterica* [35]^a^
Targetrons	Retrohoming of Mobile Group II Introns by reverse splicing and insertion in genome	*Clostridium perfringens* [38] ^b^ *Vibrio Cholera* [78] ^a^ *Yersinia pseudotuberculosis* [79] ^a^ *Staphylococcus aureus* [80]^b^
Phage Engineering CRISPR/Cas9	Delivery of CRISPR genes and RNA guides for sequence specific antimicrobials	Carbapenem-resistant Enterobacteriaceae Enterohemorrhagic *Escherichia coli* [55] ^a^
Antisense RNA	Post-transcriptional gene silencing	*Staphylococcus aureus* [81] ^b^ *Streptomyces coelicolor* [82] ^b^

^a^Gram-negative pathogens, ^b^ Gram-positive bacteria

#### Insight into bacterial virulence, resistance mechanism and biomolecular targets

Bacterial chromosomal modifications have greatly aided in better comprehension of bacterial pathogenesis and virulence mechanisms. Among the genome engineering methods, the utilization of the λ-Red recombinase system for insertions, deletions or point mutations of the genome has been very popular. Pioneered by Murphy [20] and later modified by Datsenko and Wanner [21], this method involves the introduction of single- or double-stranded DNA with chromosomal homology regions for recombination [22]. Since its conception, this editing strategy has been made more efficient by modifications to the method developed by Wanner [23–25]. This has also been readily adapted to pathogenic bacterial strains for investigating the roles of genes in pathogenesis [23, 26–29]. *Pseudomonas aeruginosa*, an opportunistic pathogen that causes nosocomial infection and chronic infections of cystic fibrosis lungs, utilizes two major quorum sensing systems LasR/LasI and RhlR/RhlI for orchestrating the production of virulence factors and biofilm formation [30]. To understand their roles in *P. aeruginosa* virulence, λ-Red recombination was successfully used to generate Δ*lasR* mutant and Δ*lasR/*Δ*rhlR* double mutant *P. aeruginosa* strains to delineate their functions [31]. Using *Caenorhabditis elegans* 24h fast-kill infection assay, it was shown that *C. elegans* was more rapidly killed by wild-type and Δ*lasR* mutant compared to the Δ*rhlR* mutant or Δ*lasR/*Δ*rhlR* double mutant strains, indicating that RhlR function is important for virulence. The authors further identified a small molecule called meta-bromo-thiolactone (mBTL), an analog of N-acyl-L-homoserine lactone (AHL), a native *P. aeruginosa* cell-cell signaling molecule, that was shown to attenuate pyocyanin and biofilm production in the wild-type strain. To identify if RhlR or LasR receptor is the molecular target for mBTL, λ-Red recombination system was used to generate Δ*lasR, and* Δ*lasR/*Δ*rhlR* double mutants to demonstrate that RhlR is the relevant in vivo target of mBTL.

One of the key adaptions of *Salmonella enterica* serovar *typhirium*, a pathogen that causes typhoid in humans, is its ability to survive inside host phagocytes. Macrophages express inducible nitric oxide (NO) synthase (iNOS) in response to lipid A, fimbriae and porins that decorate the *Salmonella* envelope. It has previously been demonstrated that NO produced as an innate response by macrophages can impact amino acid biosynthesis in *Salmonella* by targeting DksA. DksA is a key RNA polymerase regulatory protein in *Salmonella,* which has been implicated in the bacterium’s resistance. To determine if DksA is responsible for the antinitrosative defenses in *Salmonella*, Henard and Vazquez-Torres generated a *dksA* mutant using λ-Red recombination [32, 33]. They determined that Δ*dksA* mutant *Salmonella* strains are hypersusceptible to the bacteriostatic effects of NO and were noticeably attenuated in its infectivity as shown in a murine model of acute systemic infection.

In order to make genome editing high-throughput, a recombineering method called Multiplex Automated Genome Engineering (MAGE) has been developed recently to replace the conventional λ-Red recombinase based recombineering method (Fig. 1b). This method employs λ-Red recombinase system and a pool of oligos to rapidly introduce simultaneous modifications in the *E. coli* genome within days [34]. Further improvements to this method, designated pORTMAGE (portable MAGE) have been made and extended to clinically relevant strains such as *Salmonella enterica* [35]. Using pORTMAGE, ten antibiotic resistance mutations were introduced simultaneously in the genome of *S. enterica* and *E. coli* to study the extent of conservation of molecular mechanisms of antibiotic resistance amongst these bacteria.

For bacteria that are intractable to common engineering methods such as the λ-Red recombinase system, mobile group II introns have been utilized for site specific editing of the genome. Mobile group II introns are bacterial retrotransposons that contain an intron RNA and an intron-encoded reverse transcriptase. The mobile group II introns are ribozymes that can insert into specific targets by the process of retrohoming [36]. Using predictive algorithms, the intron RNA can be re-designed to form a ‘targetron’, such that a target DNA site of choice can be edited. This method has been adapted to a number of pathogenic strains for understanding mechanisms of virulence [37]. For several medically relevant *Clostridium* species which are recalcitrant to recombinations, a targetron based method named ClosTron technology has been developed for successful genome editing [38]. In one example, ClosTron technology was used for site-directed mutagenesis of a germination specific protease called CspC in *Clostridium difficile*, a causative agent of foodborne infection and diarrhea. This study helped determine the role of CspC in host bile acid recognition for in vivo germination and disease establishment [39].

In another example, the targetron technology was used in studying virulence mechanism in *Pasteurella multocida*, an animal pathogen that causes fowl cholera in wild birds and poultry, hemorrhagic septicemia in ungulates and atrophic rhinitis in swine. The polysaccharide capsule that is composed of hyaluronic acid is a major virulence factor. To investigate the mechanism of capsule formation and validate the role of global transcriptional regulator Fis in capsule formation, Steen and coworkers used the targetron technology in *Pasteurella* to generate *Fis* mutants [40]. They determined that not only is functional Fis protein required for capsule formation, but it is also required for regulation of number of virulence genes.

#### Production of novel antibiotics

The rate at which bacteria are developing resistance to existing antibiotics is alarming and warrants our immediate attention to the development of novel antibiotics for combatting bacterial pathogens. Typically, antibiotics are naturally derived small molecules that are produced by genetically encoded pathways. They represent a rich source of chemical diversity and are produced by an array of microorganisms. The use of genome editing tools to engineer new biosynthetic pathways in microbial hosts is proving to be an ideal strategy for production of novel antibiotics [41]. In one instance, Eustaquio and co-workers employed the λ-Red recombinase methodology to inactivate *clo*-*hal* and *cloz* genes in the biosynthetic gene cluster which produces the antibiotic clorobiocin, a bacterial DNA gyrase inhibitor. The mutated cosmid bearing the inactivated *clo*-*hal* cassette or *cloZ* gene was introduced in *S. roseochromogenes* to study the functional role of these genes in chlorination of the molecule. Furthermore, this strain was then used for producing an analog of clorobiocin, which has a methyl group instead of chlorine substitution and showed reduced antibiotic potency [42].

The λ-Red recombinase based recombineering method has also been applied for combinatorial biosynthesis of daptomycin (Cubicin), an antibiotic approved in the US for the treatment of skin infections caused by Gram-positive *Staphylococcus aureus* [43]*.* The antibiotic also has potent in vitro bactericidal activity against methicillin resistant *S. aureus* (MRSA), penicillin-resistant *Streptococcus pneumoniae* (PRSP), vancomycin-resistant enterococci (VRE), and vancomycin-resistant *S. aureus* (VRSA) [44]. With the emergence of bacterial resistance to this antibiotic, there has been an interest to make second generation derivatives of daptomycin. Using a novel approach, the λ-Red recombinase methodology has been used for exchanging multiple modules in the subunits of the nonribosomal peptide synthetase (NRPS) in the daptomycin biosynthetic pathway using *E. coli* as a heterologous host [45]. The combinatorial biosynthesis approach was used to generate a library of novel lipopeptides with modifications of the core peptide, of which some compounds were as active as daptomycin. The above examples illustrate the huge potential of bacterial genome engineering tools in biosynthesis of novel antibiotics.

#### Attenuated vaccine strain development

One of the effective strategies to prevent infections is the use of vaccination, which establishes an immunological memory of a foreign agent by triggering the body’s innate immune response. Inactivated or attenuated vaccines from the actual pathogen have been successfully used in the past for defense against bacterial infections. Attenuated live vaccines are created by decreasing the virulence of the pathogen to weaken their infection potential without compromising the robust host immune response that is required for protection during future infections that could be caused by the same pathogen (For example: MTBVAC against *Mycobacterium tuberculosis* [46], Ty21a against *Salmonella typhi* [47]). There is a growing focus on using recombinant DNA technology for producing attenuated strains of pathogenic bacteria [48]. Genome engineering tools are now being routinely explored for the possibility of reducing bacterial virulence. For example, Ranallo and coworkers have successfully demonstrated the extension of the λ- Red recombination method in *Shigella* for the development of live vaccine strains [49]. They utilized over 13 different isogenic strains to delete genes involved in various functions such as intracellular growth and survival *(asd),* cell to cell spread (*virG*), invasion (*ipaB*), enterotoxic activity (*set1A*, *sen*) to name a few. Utilizing a plaque assay, they determined that the *virG* deleted strain reduced plaque formation significantly. In further virulence testing using the keratoconjuctivitis model (Sereny test), they found that only the *virG* deleted strain was attenuated.

More recently, Salehi and cowokers validated the utilization of this recombineering technique for the production of live attenuated *Shigella dysenteriae* strain by deleting *ipaD* gene [50]. The ipaD is a chaperonin protein and part of the type III secretion system which secretes invasion plasmid antigen (Ipas) proteins that are responsible for *Shigella* penetration and invasion into epithelial cells. The authors hypothesize that the deletion of *ipaD* gene could potentially inhibit secretion of IpaD, IpaB and IpaC proteins and thereby suppress *Shigella* invasion.

The targetron methodology has also been used to generate candidate vaccine strains in pathogens where the λ- Red recombination method works poorly. Combining the targetron methodology discussed above and the well-known Cre-lox recombinase system, a vaccine strain of the Gram-positive pathogen *Staphylococcus aureus* was generated by a novel approach called Genome Editing via Targetrons and Recombinases (GETR) [37]. The researchers generated introns that could integrate *lox* sequences upstream and downstream of the 15-kb *Staphylococcus aureus* pathogenicity island I (SaPI-1). The *lox* sites are specific DNA sequences that can be targeted by the Cre recombinase enzyme. The expression of Cre recombinase resulted in Cre-mediated recombination that deleted the intervening region in the SaPI-1 leading to the generation of a vaccine strain. The application of such techniques in clinical isolates of *Staphylococcus* can be very useful in generating vaccine strains for MDR strains for overcoming the antibiotic resistance challenge. Though the safety and efficacy of these vaccines need to be assessed periodically, the above examples highlight the power of genome editing tools for their potential to design live vaccines. Development of promising and safe to use vaccines will have broad applications in preventive healthcare and will be a stepping stone for the development of oral vaccines in other pathogenic bacteria [51].

#### Specificity in pathogen killing and pathogen detection for diagnosis

Tools adapted from bacteriophage assist in understanding host-pathogen interactions and serve as targeted therapeutics [52–54]. Phage can specifically kill virulent strains of bacteria that bear very close sequence alignment to harmless strains via the use of the clustered regularly interspaced short palindromic repeats (CRISPR)/Cas9 system. The CRISPR/Cas9 system specifically targets a DNA sequence for double strand break formation, resulting in death of the bacteria. Phage can deliver RNA guides with CRISPR associated proteins (Cas) to pathogenic bacteria [55, 56].

Towards targeted therapies against pathogenic bacteria, Citorik et al. have demonstrated sequence specific antimicrobials [55], which overcome extremely high genome sequence similarity between non-pathogenic and pathogenic strains by targeting small sequence variations present in the pathogenic strain. It is possible to harness the ability of Cas to target specific sequences and differentiate between a little as one mismatch between target and non-target genomic DNA to kill the pathogenic bacterial population [55]. In the model system of *Galleria mellonella* larvae, targeted nucleases showed greater antimicrobial activity than antibiotic chloramphenicol. The RNA guided DNA nucleases targeting of the enterohemorrhagic *E. coli* intimin virulence gene and the nuclease activity at this locus proved toxic to the pathogenic bacteria [55].

In another application of the CRISPR system, Yosef et al. designed a novel two phage CRSIPR system consisting of temperate and lytic phage programmed to specifically sensitize and kill antibiotic resistant bacteria [57]. Initially, lysogenic phage carrying CRISPR machinery was used to target the antibiotic resistance genes and confer lytic phage resistance to these cells. The cells became sensitive to antibiotic but were resistant to lytic phage. In the second step, lytic phage was used to kill any remaining antibiotic resistant cells thus enriching the population of the antibiotic sensitive cells, which can then be killed with antibiotics. The authors propose that this strategy can be very useful for treating hospital surfaces or for skin surfaces of medical personnel.

Phage can also serve as diagnostic and detection tools for infection yet do not require amplification of the host bacteria as the phage population increases during infection. As the phage infects specific bacteria, the phage genomic template becomes enriched in the population and targeted bacteria can be killed by the phage. A quantitative PCR following phage infection can indicate amplification of phage DNA infecting a specific bacteria [53]. Diagnostic phages exist for highly pathogenic bacteria, such as phage phi A1122 for *Yersinia pestis*, as well as phage reporter systems for *Bacillus anthracis* and *Mycobacterium tuberculosis* [58].

### Application of synthetic biology for targeting bacterial infections

**Fig. 2:**
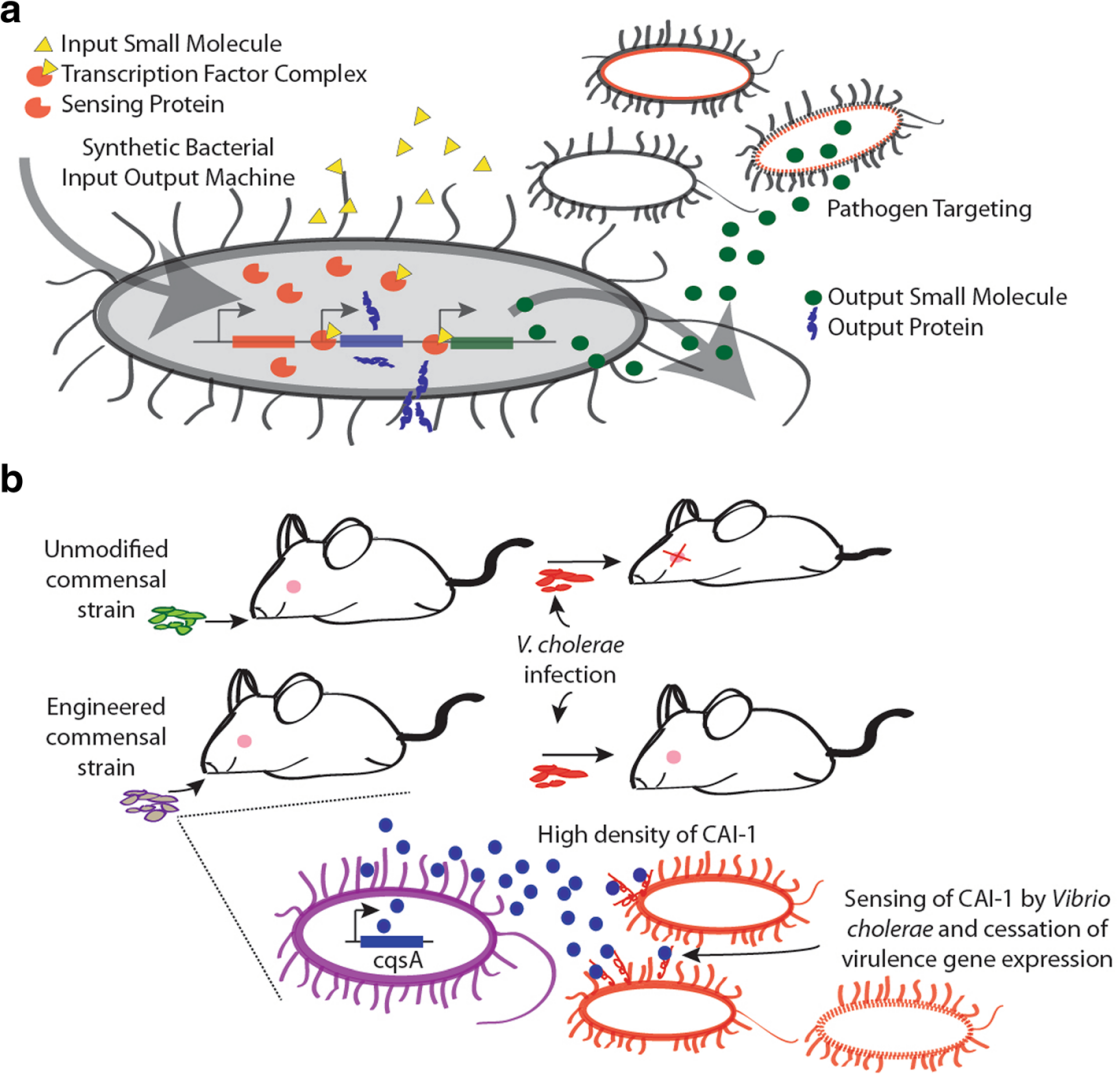
**a**) Synthetic biology circuits can be engineered with desired input and output signals to kill bacterial pathogens; **b**) Engineering probiotic bacteria that expresses QS molecule Cholerae autoinducer-1 (CAI-1) to target *Vibrio cholerae* infection in a mouse model [65]

Key advances in precision genome engineering have resulted in the development of a toolbox that is proving highly valuable for redesigning microbial genome structure for useful applications. The bacterial genome engineering strategies discussed in the previous sections clearly illustrate the utility of these tools in synthetic biology applications for targeting infectious diseases. Apart from gene insertions, deletions or mutations for modification of the genome using engineering tools, one of the main goals of SB is to build and integrate gene circuits which process signals within a living cell for a desired output. Gene circuits have been assembled in microbes using biological parts or functional units for various biomedical applications [59, 60]. Modular biological parts can be connected to develop circuits based on electrical engineering principles with input and output responses that can be analog or digital. Using this engineering framework, SB has potential applications in biofuel production, synthesis of industrial chemicals or natural product substitutes, biomedical applications or understanding and countering bacterial infections (Fig. 2a) [2, 5, 59]. In the following section, we highlight few examples of regulatory biological components that have been utilized for biomedical applications focused on bacterial infections.

#### Bacterial quorum sensing based circuits

Bacteria effectively sense and respond to environmental signals as part of their natural survival and proliferation strategies. SB has harnessed these mechanisms to sense and respond to clinically relevant signals. The development and engineering of bacterial small molecule signaling mechanism based SB circuits for targeting bacterial pathogens has also been used as a novel approach in design of circuits.

Bacterial cell population-density dependent behavior termed quorum sensing (QS) is a highly evolved natural signaling circuit found in bacteria [60]. It involves small molecule signal production and sensing by the native bacterium via their cognate signal receptor that then modulate the expression of the target genes [60]. Initial QS SB circuit engineering in bacteria was demonstrated by Weiss and Knight Jr. [61]. One of the *Vibrio fischeri* QS systems relies on a key small molecule signal N-Acyl-L- homoserine lactone (AHL) for its bioluminescence production. In their first SB circuit, AHL synthesis catalyzed by luxI (AHL synthase) and the AHL signal receptor luxR were engineered into two separate populations (A and B populations respectively) of *E. coli.* When these strains were cocultivated, it was observed that the AHL signals produced by *E. coli (luxI)* (population A) freely diffused out the cell and bound to its cognate LuxR receptor in *E. coli (luxR)* subpopulation (population B). The activated LuxR-AHL complex in turn activated *luxI::gfp* promoter-reporter fusion resulting in GFP production [61]. With this early demonstration, interest in the utility of QS circuits in SB has increased in a number of biomedical applications including diagnostic tools, cancer, immune diseases, metabolic disorders, infectious disease therapies, drug production through fermentation, biosensing, etc [62–64].

In an important example, Duan and March showed that feeding infant mice with engineered probiotic *E. coli* to constitutively overexpress *Vibrio cholerae* QS signal (*S*)-3-hydroxytridecan-4-one or Cholerae autoinducer-1 (CAI-1) that down-regulates biofilm production substantially increased the mice survival rate from *V. cholerae* infections (92% with 8h pretreatment) [65] (Fig. 2b). Using a gene circuit, Saeidi et al. engineered *E. coli* with *P. aeruginosa* LasR AHL receptor to sense *P. aeruginosa* AHL signal N- 3-oxo-dodeconoyl-L-HSL and auto-regulate the activation of killing and lysis gene products (E7 lysis protein and Pyocin S5) that targeted *P. aeruginosa* [66]. In conjunction with this design, other QS circuits for pathogen targeting based on detection, destruction and secretion modules have been successfully engineered into useful bacteria. For example, in a proof-of-concept study, pathogen *P. aeruginosa* elimination was demonstrated using programmed *E. coli* sensing *P. aeruginosa* QS signal N- 3-oxo-dodeconoyl-L -HSL and activating the production and secretion of chimeric lethal protein bacteriocin CoPy [67].

Another example of this modular bacterial QS circuit engineering was demonstrated by programming *E. coli* to seek and kill *P. aeruginosa*. This system utilized CheZ, a motility promoting protein and two engineered secrete and kill proteins DNaseI and MicrocinS that promoted biofilm disruption and lethality [68]. Most of these QS circuits essentially target Gram-negative bacterial circuits and in particular only the AHL class of QS molecules. There is a great diversity of QS molecules (non-AHL classes) and pathways that still remain underexplored for targeting and can be effectively used for pathogen control.

#### RNA based circuits

RNA based biological parts called riboregulators are also gaining attention in SB circuit design due to their tunable and modular nature. These RNA hairpin tools are designed to sequester the ribosome binding site (RBS) upstream of the start site of the mRNA encoding a gene in order to block translation [58, 69–72]. In one application of these tools, RNA switches called “toehold” switches developed by Pardee et al. have been used for diagnostic application for in vitro, cell-free, paper-based devise for sensing Ebola mRNA and mRNAs of antibiotic resistance genes [73]. RNA based diagnostic gene network consists of a reporter gene network (*gfp*, *lacZ*), where the RBS is sequestered upstream by the toehold switch. The gene network and a cell-free coupled transcription/translation system are freeze dried on a paper or other porous material and can be activated by rehydration with the test sample which consists of the mRNA to be detected. Messenger RNA sensors for antibiotic resistance genes, upon sensing the target gene, showed significant induction of reporter gene making this tool highly promising and cost-effective in the detection and diagnosis of bacterial infections in clinical samples.

**Fig. 3:**
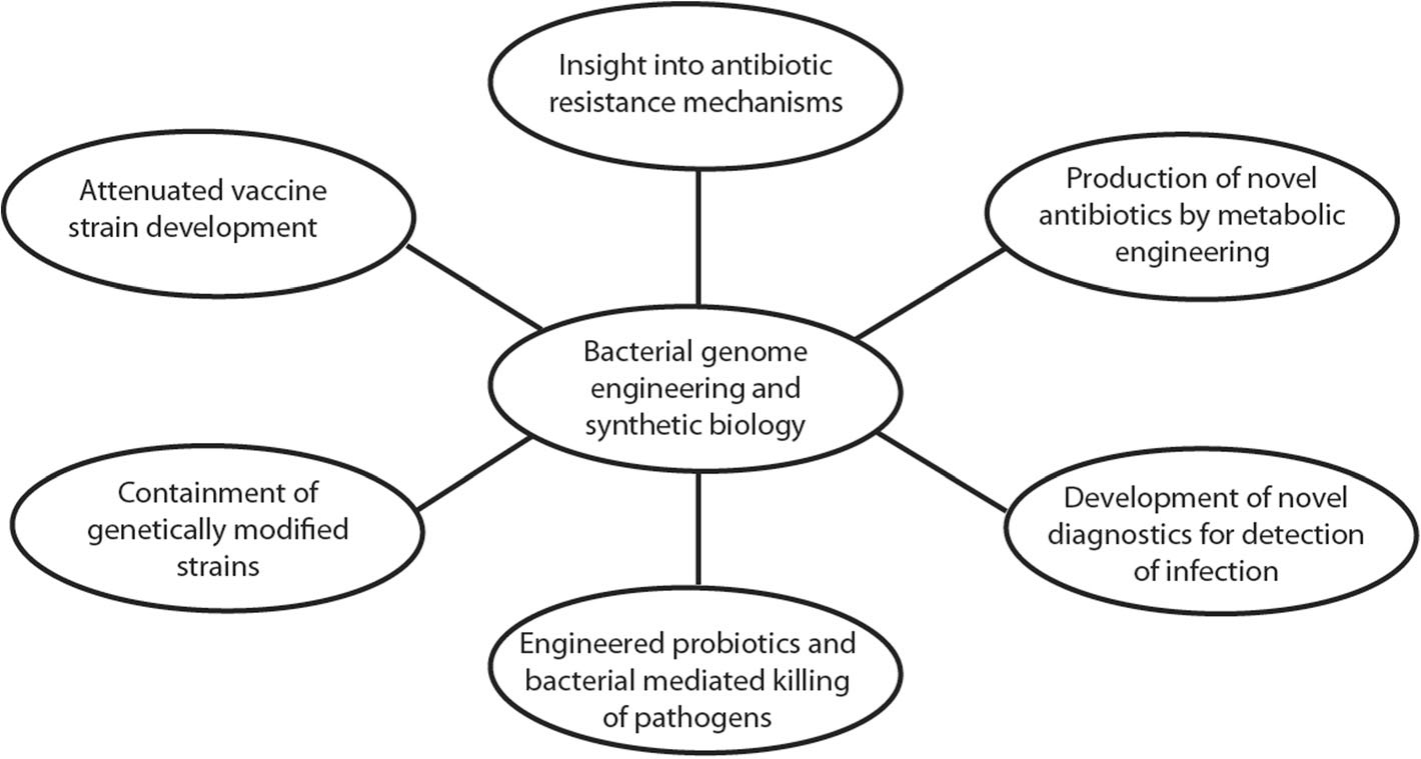
Multipronged use of SB and genome engineering tools to counter bacterial infections

Apart from application in diagnostics, the ability of fine-tuning gene expression and the modular nature of riboregulators make them excellent candidates for chromosomal integrations using existing genome editing tools for biosynthetic pathway engineering in bacterial hosts. It is evident from the above examples that genome engineering and synthetic biology tools in bacteria can have significant impact on a number of applications for targeting bacterial infections (Fig. 3).

## Conclusions

The integration of engineering principles and biology in the last decade has opened up new avenues for development of novel therapeutics in treating diseases. The rapid emergence of bacterial antimicrobial resistance and limited success in identifying new antibiotics warrants the identification and validation of novel bacterial drug targets. SB driven genome editing techniques offer new avenues to pursue bacterial target identification and present the possibilities for development of novel antimicrobial therapeutics. The availability of high-throughput bacterial genome editing tools coupled with advancement in DNA synthesis technologies provides new opportunities for metabolic engineering of large gene clusters in microbial hosts. By rational combination of gene parts, biosynthesis can be potentially reprogrammed for generation of novel small molecules for therapeutic purposes [48]. This is further facilitated by *in silico* whole genome mining and software algorithms that predict the gene clusters which can be used in biosynthetic pathway engineering for production of novel antibiotics [48, 74]. This offers huge potential for combinatorial biosynthesis of natural product analogs for the discovery of novel antibiotics, as in the example of antibiotic daptomycin described in the earlier section [41].

In addition to antibiotics, development of alternative treatment options employing bacteriophage and probiotic bacterial engineering for pathogen targeting and destruction are critical for the fight against infections, including the drug-resistant bacterial pathogens. Advances in computational gene circuit design coupled with improvements in large scale DNA synthesis heralds a new era of SB based therapeutic approaches. It is now possible to design whole bacterial genomes from synthesized components. Viewing genetic code as an analog to computer software, one can then “boot” the synthetic genome in a compatible cellular environment [75]. Additionally, computational design of synthetic circuits will be highly useful for predicting the optimal combination of synthetic parts for a desired cellular function. Recently, Voigt and coworkers developed the Cello software, which allows a user to program a desired circuit function in *E. coli* and compile the code into a DNA sequence for synthesis [76]. Computer-aided design (CAD), high-throughput DNA synthesis and advanced genome editing techniques will prove highly valuable for generating re-programmed bacterial strains for therapeutic applications. The combination of advanced computational methods for predictive SB systems, and rapid progress in technologies for efficient large scale DNA synthesis as well as high-throughput, automated genome engineering such as MAGE, pORTMAGE indicates that SB has immense potential to deliver novel solutions for pathogen control.

Genetically modified bacteria offer great hope for finding novel solutions to detect and treat infections. However, it is also imperative to periodically asses the biosafety of these organisms to avoid accidental release of synthetic bacteria generated in some of these applications. To address this concern, two engineered safeguard systems called the ‘Deadman’ and ‘Passcode’ kill switches have been developed by Collins and colleagues in *E. coli* [77]. These switches are based on circuits that need specific input(s) of small-molecules for cell survival. In the absence or presence of the specific molecules, toxin gene expression is activated leading to cell death. The kill switch circuit designs can potentially be incorporated in a broad range of bacterial hosts to ensure safe handling of these modified organisms. It is clearly evident from the above examples that SB is bridging the gap between basic and translational research. It is expected that with continuous technological advances in this field and with development of new programmable biological tools, SB has enormous potential to develop biomedical therapies to prevent and treat diseases caused by bacterial infections.

## Abbreviations

AHL: N-acyl-L-homoserine lactone
CAI-1: Cholerae autoinducer-1
CDC: Centers for disease control and prevention
CRISPR: Clustered regularly interspaced short palindromic repeats
DNA: Deoxyribonucleic acid
GETR: Genome editing via targetrons and recombinases
GFP: Green fluorescent protein
iNOS: inducible nitric oxide synthase
MAGE: Multiplex automated genome engineering
mBTL: meta-bromo-thiolactone
MDR: Multidrug resistant
mRNA: messenger ribonucleic acid
MRSA: Methicillin resistant *Staphylococcus aureus*
NO: Nitric oxide
NRPS: Nonribosomal peptide synthetase
PCR: Polymerase chain reaction
pORTMAGE: portable multiplex automated genome engineering
PRSP: penicillin-resistant *Streptococcus pneumoniae*
QS: Quorum sensing
RBS: Ribosomal binding site
RNA: Ribonucleic acid
SB: Synthetic biology
VRE: Vancomycin-resistant enterococci
VRSA: Vancomycin-resistant *Staphylococcus aureus*
WHO: World Health Organization
